# P-1756. Utility and Clinical Impact of Karius Testing in the Adult Population of a Tertiary Care Center: A Retrospective Study

**DOI:** 10.1093/ofid/ofaf695.1927

**Published:** 2026-01-11

**Authors:** Jillian S Catalano, Drew W Charles, Alexandra G Mills, Scott R Curry, Ruth O Adekunle, Yosra Alkabab, Charles Teixeira, Rachel Burgoon, Logan Patterson, Eric G Meissner, Divisha Sharma, Susan E Dorman, Courtney E Harris

**Affiliations:** Medical University of South Carolina, Charleston, SC; Medical University of South Carolina, Charleston, SC; Medical University of South Carolina, Charleston, SC; Medical University of South Carolina, Charleston, SC; Medical University of South Carolina, Charleston, SC; MUSC, Charleston, South Carolina; Medical University of South Carolina, Charleston, SC; Medical University of South Carolina, Charleston, SC; MUSC, Charleston, South Carolina; Medical University of South Carolina, Charleston, SC; Medical University of South Carolina, Charleston, SC; Medical University of South Carolina, Charleston, SC; Medical University of South Carolina, Charleston, SC

## Abstract

**Background:**

Karius testing (KT) utilizes cell-free DNA metagenomic next-generation sequencing to detect infectious organisms in plasma samples. More data on the clinical impact of KT in large cohorts are needed to understand its utility and relevance in different clinical syndromes. We examined the utilization, patient clinical characteristics, and clinical impact of KT in a retrospective cohort of adults at a large tertiary care center.

**Figure:**
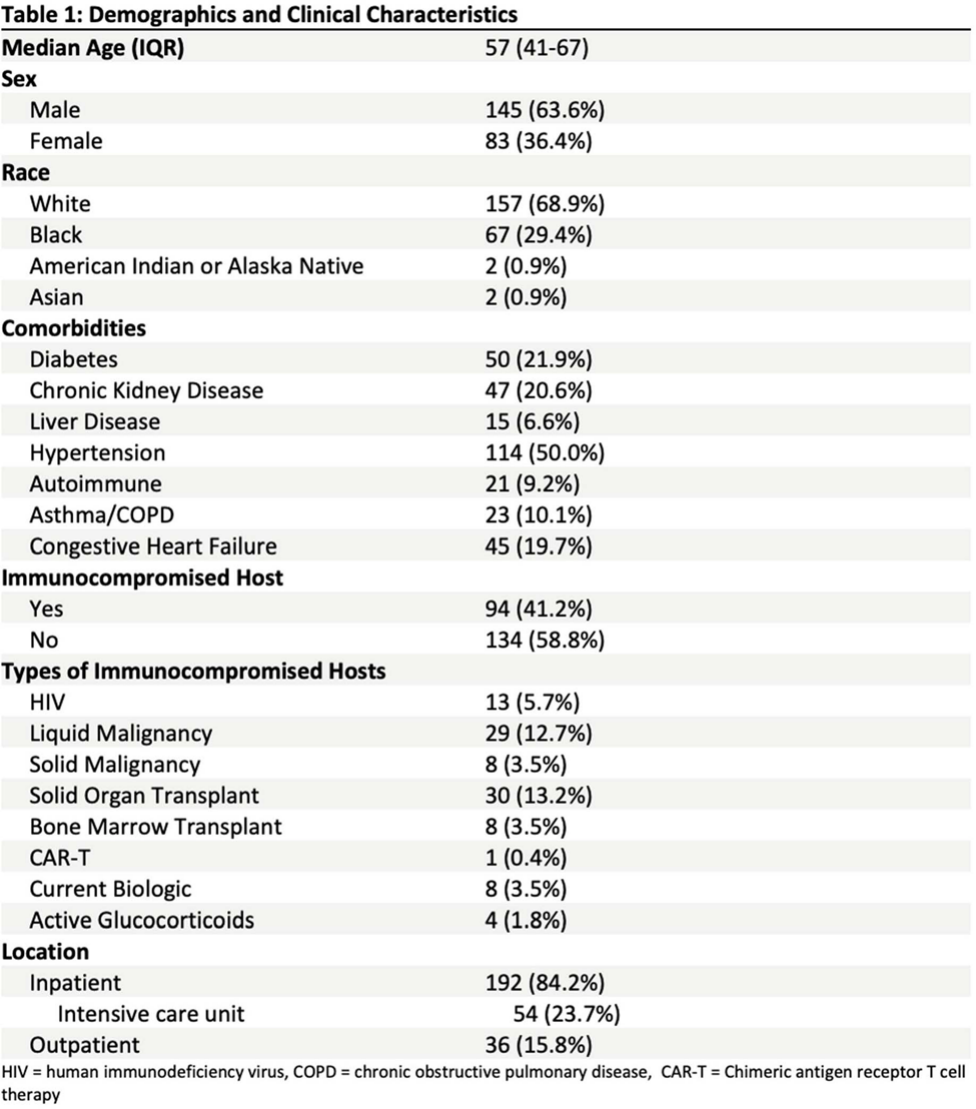

**Methods:**

Retrospective review of all KT completed for adult patients at the Medical University of South Carolina from January 2022 to November 2024. Clinical impact (positive, negative, or neutral) was assessed using pre-specified criteria. Descriptive analyses of microorganisms identified, patient characteristics, and clinical impact were performed.

**Figure:**
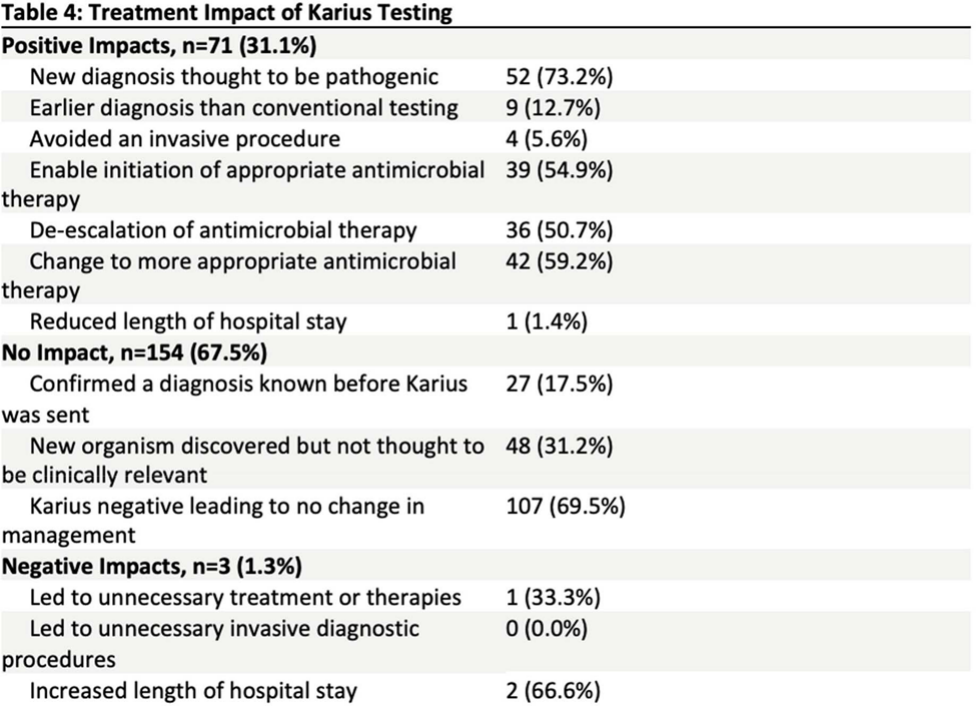

**Results:**

228 patients had a KT; 94 (41%) were immunocompromised (Table 1). Main indications for testing were to establish a diagnosis of a problem presumed to be infectious (n=149, 65%) and to exclude infection (n=72, 32%) (Table 2). KT was positive in 123 (54%) patients. The most frequent organisms detected are outlined in Table 3. Mean time from KT collection to result was 4.57 days. KT had a positive clinical impact in 71 (31%) patients, mainly through identification of a microorganism considered by clinical care providers to be pathogenic (n=52). KT had a neutral clinical impact in 154 (68%) patients, mainly because negative KT results did not lead to changes in patient management (n=107). There was a negative clinical impact due to KT in 3 (1%) patients, including 2 patients with increased length of hospital stay (Table 4). In 60 (26%) patients, KT identified an organism when standard testing was negative. Standard testing and KT were concordantly positive in 35 (15%) patients and concordantly negative in 105 (46%) patients. In 17 (8%) patients, KT returned negative when standard testing was positive.

**Conclusion:**

Among adults with a wide variety of clinical syndromes, KT had a positive clinical impact in nearly one-third, with rare negative impact. In over one-quarter of tested adults, KT identified an organism that was not discovered with standard testing. Further studies should seek to identify clinical syndromes where KT has the highest likelihood of clinical utility.

**Disclosures:**

Rachel Burgoon, Pharm.D., Merck: Grant/Research Support

